# lncRNA HAGLR modulates myocardial ischemia–reperfusion injury in mice through regulating miR-133a-3p/MAPK1 axis

**DOI:** 10.1515/med-2022-0519

**Published:** 2022-07-19

**Authors:** Zi Wang, Wenqi Luo, Peng Zhong, Yifan Feng, Huaibin Wang

**Affiliations:** Department of Cardiosurgery, Beijing Hospital, National Center of Gerontology, Institute of Geriatric Medicine, Chinese Academy of Medical Sciences, No. 1 Da HuaRoad, Dongdan, Beijing, China; Department of Cardiosurgery, Beijing Hospital, National Center of Gerontology, Institute of Geriatric Medicine, Chinese Academy of Medical Sciences, Dongdan, Beijing, China

**Keywords:** myocardial ischemia–reperfusion injury, HAGLR, miR-133a-3p, MAPK1

## Abstract

Acute myocardial infarction is one of the leading causes of morbidity worldwide, but the underlying mechanism responsible for myocardial ischemia–reperfusion (I/R) injury remains elusive. lncRNA plays roles in inflammatory response, cell apoptosis and regulation of myocardial ischemia. However, whether lncRNA HAGLR could regulate myocardial I/R injury and the molecular mechanism need to be further investigated. lncRNA has been shown to bind to miRNAs and compete with endogenous RNAs. miR-133a-3p has been shown to regulate cardiomyocyte apoptosis and ischemic myocardial injury. In this work, it has shown that knockdown of HAGLR could suppress inflammatory response and cell apoptosis induced by I/R and, thus, alleviate myocardial I/R injury. HAGLR promoted myocardial I/R injury by inhibiting miR-133a-3p to promote MAPK1 expression.

## 1 Introduction

Acute myocardial infarction remains a leading cause of morbidity worldwide despite great progress has been made in promoting the prognosis recovery of patient over decades [1]. Early myocardial reperfusion, combined with the use of thrombolytic therapy and primary percutaneous coronary intervention, is effective to attenuate myocardial infarct size and improve clinical outcome when acute myocardial infarction occurs [2]. However, the reperfusion of ischemic myocardium can induce myocardial reperfusion injury [2]. Nevertheless, the underlying mechanisms responsible for myocardial ischemia–reperfusion (I/R) injury are complicated and elusive. Therefore, myocardial I/R injury remains an unsolved clinical issue, which needs more studies to uncover new therapeutic targets.

Non-coding RNAs are basically composed of two categories including microRNA (miRNA) with a length of about 22 nt and long non-coding RNA (lncRNA) with a minimum size of 200 nt [3,4,5,6,7]. lncRNA is a kind of transcripts that do not encode proteins, distributed in the nucleus and cytoplasm [7]. lncRNA plays a role in mediating gene expression via many pathways, such as epigenetic modification, RNA stability and chromosomal elimination and translocation [8]. Thus, lncRNA has been reported to be involved in the pathophysiological process of various types of tumors, including proliferation, invasion, metastasis and apoptosis [9,10,11,12]. lncRNA has been demonstrated to play a role in the regulation of myocardial ischemia. lncRNA PEAMIR can compete with miR-29b-3p and alleviate apoptosis and inflammatory response in myocardial I/R aggravated by PM2.5 exposure [13]. lncRNA CAIF suppresses autophagy and alleviates myocardial infarction by preventing myocardin transcription mediated by p53 [14]. lncRNA enhances inflammatory response and cell apoptosis and aggravates neuropathic pain through activating NLRP3 inflammasome via miR-182-5p/ATAT1 axis [15]. However, the function and mechanism of HAGLR regulating myocardial injury induced by I/R have not been reported yet.

Deregulated miRNAs have been implicated in a variety of diseases [16]. Emerging reports demonstrated that lncRNA acted as competitive endogenous RNAs and released mRNAs by binding to miRNAs, involving in modulating gene expression at the post-transcriptional level [15]. miR-133a-3p belongs to the miR-133 family. It has revealed that miR-133a-3p exhibited multiple functional roles in various physiological processes, such as myoblast proliferation and differentiation, malignancies [17]. miR-133a-3p is frequently identified to be downregulated in some types of malignancies, indicating that miR-133a-3p may serve as a diagnostic indicator of carcinoma [17]. miR-133a-3p has also demonstrated to be involved in cardiomyocyte apoptosis and ischemic myocardial injury [18].

In this study, HAGLR was found to be upregulated in the myocardial tissue of mice with I/R injury. Knockdown of HAGLR alleviated myocardial I/R injury and inhibited inflammatory response. HAGLR modulated MAPK1 expression by targeting miR-133a-3p, thus regulating myocardial injury induced by I/R.

## 2 Materials and methods

### 2.1 Animals and generation of I/R model

Adult male rats were purchased from Beijing HFK Bioscience Co. Ltd. Experimental protocols were approved by the Beijing Hospital, National Center of Gerontology, Committee of Experimental Animals. Pentobarbital sodium was intraperitoneally injected to anesthetize rats. The chest was opened at the fourth intercostal space of the left subclavian midline to expose the heart. The left anterior descending coronary artery was ligated for 30 min and restored myocardial perfusion was performed for 2 h to induce I/R. The blood was collected for analysis. The same surgical procedures were conducted in the sham group except ligation of the left anterior descending coronary artery. HAGLR was knocked down through injecting adeno-associated virus expressing HAGLR-specific shRNA into rat hearts.

### 2.2 Human cell lines and reagents

H9C2 rat cardiomyoblast cells were purchased from ATCC (Manassas, VA, USA). H9C2 cells were cultured in DMEM supplemented with 10% fetal bovine serum (FBS), 100 mg/mL streptomycin and 100 U/mL penicillin in a humidified atmosphere supplemented with 5% CO_2_ at 37°C. FBS and trypsin-EDTA were purchased from Thermo Fisher Scientific. LDH assay kit (ab102526) and TUNEL assay kit (ab66110) were purchased from Abcam. Antibodies against MAPK1 (TA500485), β-actin (AM4302) and HRP-labelled secondary antibody were purchased from Thermo Fisher Scientific.

### 2.3 Hypoxia and reoxygenation

Cells were cultured in a container with sealed air in an anaeropack, which generated a hypoxic atmosphere and cells were maintained for about 18 h at 37°C. Then, the anaeropack was removed and cells were incubated for 6 h at 37°C in a humidified 5% CO_2_ atmosphere.

### 2.4 Production of lentiviral vector for HAGLR knockdown

PEI-based method was used for the transfection and generation of recombinant lentiviruses in HEK293T cells. Lentiviruses were harvested at 48 h post-transfection and filtered with 0.45 μm cellulose acetate filters. Cells were infected with the lentiviruses and selected with puromycin for the generation of HAGLR-knockdown cell line.

### 2.5 Real-time quantitative PCR (qRT-PCR)

Total RNA was extracted with TRIzol (Invitrogen). SYBR Green (Takara, Japan) was used to prepare the reaction mixture, and ABI PRISM 7500 RT PCR instrument was used for detection. The relative mRNA expression level was quantified with 2^−ΔΔCt^ method. β-Actin acted as the internal control. The sequence of primers used were as follows: HAGLR-forward: 5′-GGGCTGGTACAGACTAGGGA-3′ and HAGLR-reverse: 5′-TAAGCAGGTCAGAAAGGGCG-3′; miR-133a-3p-forward: 5′-CAATGCTTTGCTAAAGCTGG-3′ and miR-133a-3p-reverse: 5′-TCAATGCATAGCTACAGCTGG-3′; β-actin-forward: 5′-CATGTACGTTGCTATCCAGGC-3′ and β-actin-reverse: 5′-CTCCTTAATGTCACGCACGAT-3′; and MAPK1-forward: 5′-AAGCGCCATTCAAGTTTGACA-3′ and MAPK1-reverse: 5′-GGCTGGAATCTAGCAGTCTCTTC-3′.

For the generation of HAGLR-knockdown cell line, the primers used to generate into pLKO.1-Puro vector were as follows: sh-NC-sense strand: 5′-ACTGCCCTGATGCTAGCTAGCACCGGT-3′ and sh-NC-antisense strand: 5′-GCUCGATCCTGCTAGATCUUCGCUAC-3′; sh-HAGLR-sense strand: 5′-GGUCGAUUGAUUGCAUCUATT-3′; and sh-HAGLR-antisense strand: 5′-UAGCUC UAACCAGAGACAUTT-3′.

### 2.6 2,3,5-triphenyltetrazolium chloride (TTC)

The heart was perfused with 4% TTC (Sigma) and fixed in 8% formalin. Then, the heart was maintained at −20°C for about 20 min. And the heart was cut into sections.

### 2.7 ELISA

The levels of IL-6, IL-1β and TNF-α were detected with ELISA kits (eBioscience, USA) following the manufacturer’s instructions.

### 2.8 TUNEL assay

The assay was conducted with Promega TUNEL assay kit (cat no. G3250). Myocardial tissue was embedded into paraffin. Then, they were deparaffinized and rehydrated with ethanol. The tissue was fixed in 4% paraformaldehyde and treated with the rTDT incubation buffer. Finally, the sample was counterstained with DAPI and photographs were captured with a fluorescence microscope (Nikon).

### 2.9 Luciferase assay

Cells were harvested by centrifugation at 600×*g* for 5 min. Cells were washed twice with cold PBS and resuspended in reporter lysis buffer. Cells were incubated on ice for 20 min and centrifuged at 1,200×*g* for 20 min. The supernatant was collected and protein concentration was quantified by the BCA assay. Then, luciferase assay reagent was added into the supernatant. A luminometer was used to detect the fluorescence.

### 2.10 Western blotting

Myocardial tissue or H9C2 cells were lysed in the RIPA lysis buffer supplemented with protease inhibitor cocktail. The supernatants were collected and protein concentration was quantified by the BCA assay. The protein was separated by SDS-PAGE, followed by immunoblotting. Blots were visualized with an enhanced chemiluminescence.

### 2.11 Statistical analysis

Data were presented as mean ± standard error of the mean with at least three independent experiments. The statistical significance was analyzed with one-way analysis of variance. *p* < 0.05 was considered as statistically significant difference.


**Ethical approval:** Ethical approval was obtained from the Ethics Committee of Beijing Hospital, National Center of Gerontology.

## 3 Results

### 3.1 Knockdown of HAGLR alleviated myocardial I/R injury

**Figure 1:**
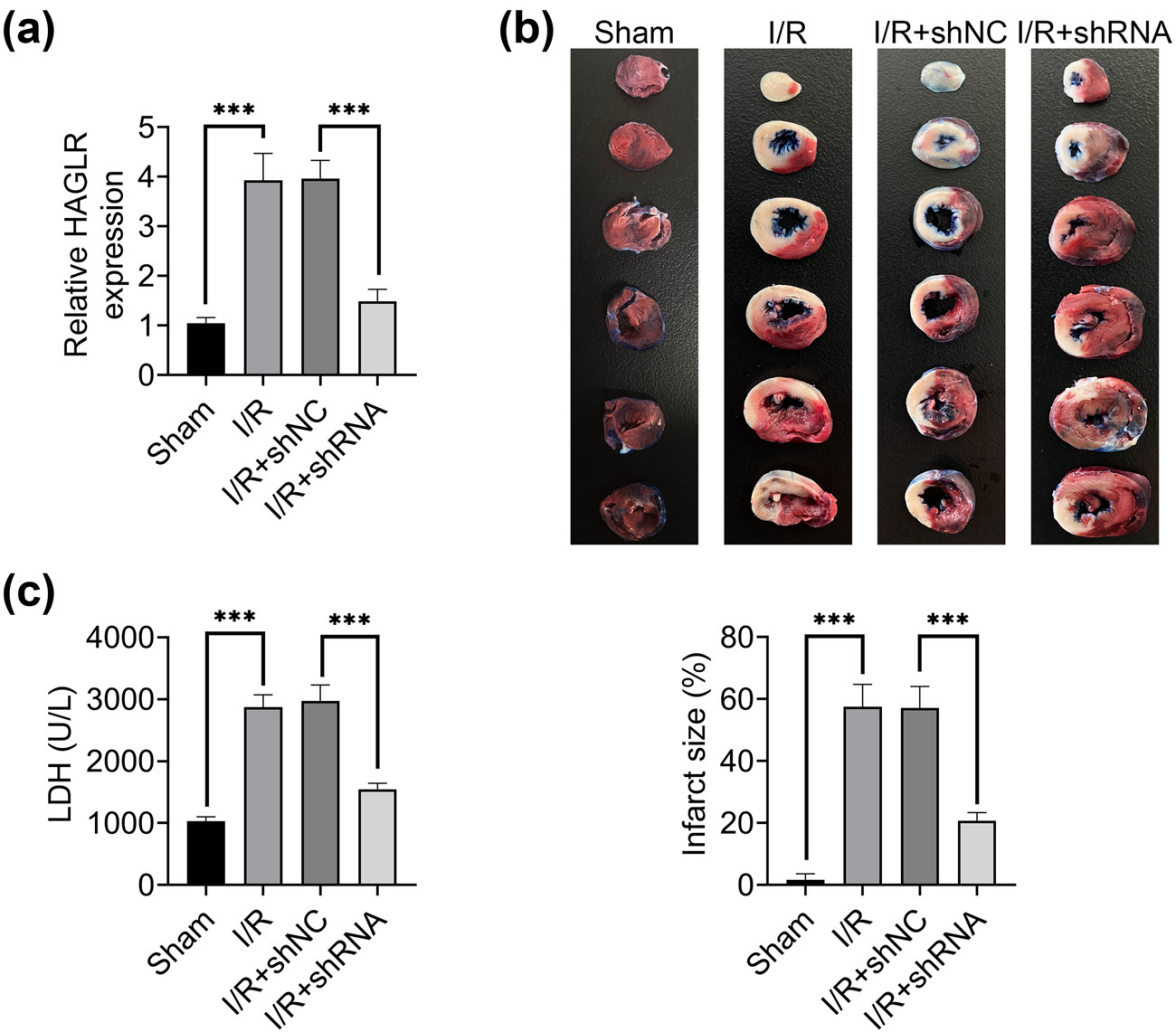
Knockdown of HAGLR alleviated myocardial I/R injury. (a) The expression level of HAGLR in I/R mice (I/R) and HAGLR-knockdown mice (I/R + shRNA) was assessed by qRT-PCR. ^***^ *p* < 0.001. Data were expressed as mean ± SD of three independent experiments. (b) *Upper*: representative photos of myocardial infarct in I/R and I/R + shRNA groups after TTC staining. *Lower*: quantification of myocardial infarct size in I/R and I/R + shRNA groups. ^***^ *p* < 0.001. Data were expressed as mean ± SD of three independent experiments. (c) The LDH level in carotid arterial blood in I/R and I/R + shRNA groups. ^***^ *p* < 0.001. Data were ex*p*ressed as mean ± SD of three independent experiments.

The expression level of HAGLR was dramatically increased in I/R and significantly decreased by shRNA technique (Figure 1a). TTC staining revealed that infarct size was significantly enlarged by I/R, which was prevented by the knockdown of HAGLR (Figure 1b). The LDH level in carotid arterial blood was significantly increased in mice with myocardial I/R injury, which was also inhibited by the knockdown of HAGLR (Figure 1c). These data suggested that knockdown of HAGLR could alleviate myocardial I/R injury.

### 3.2 Knockdown of HAGLR inhibited inflammation and cell apoptosis induced by I/R

**Figure 2:**
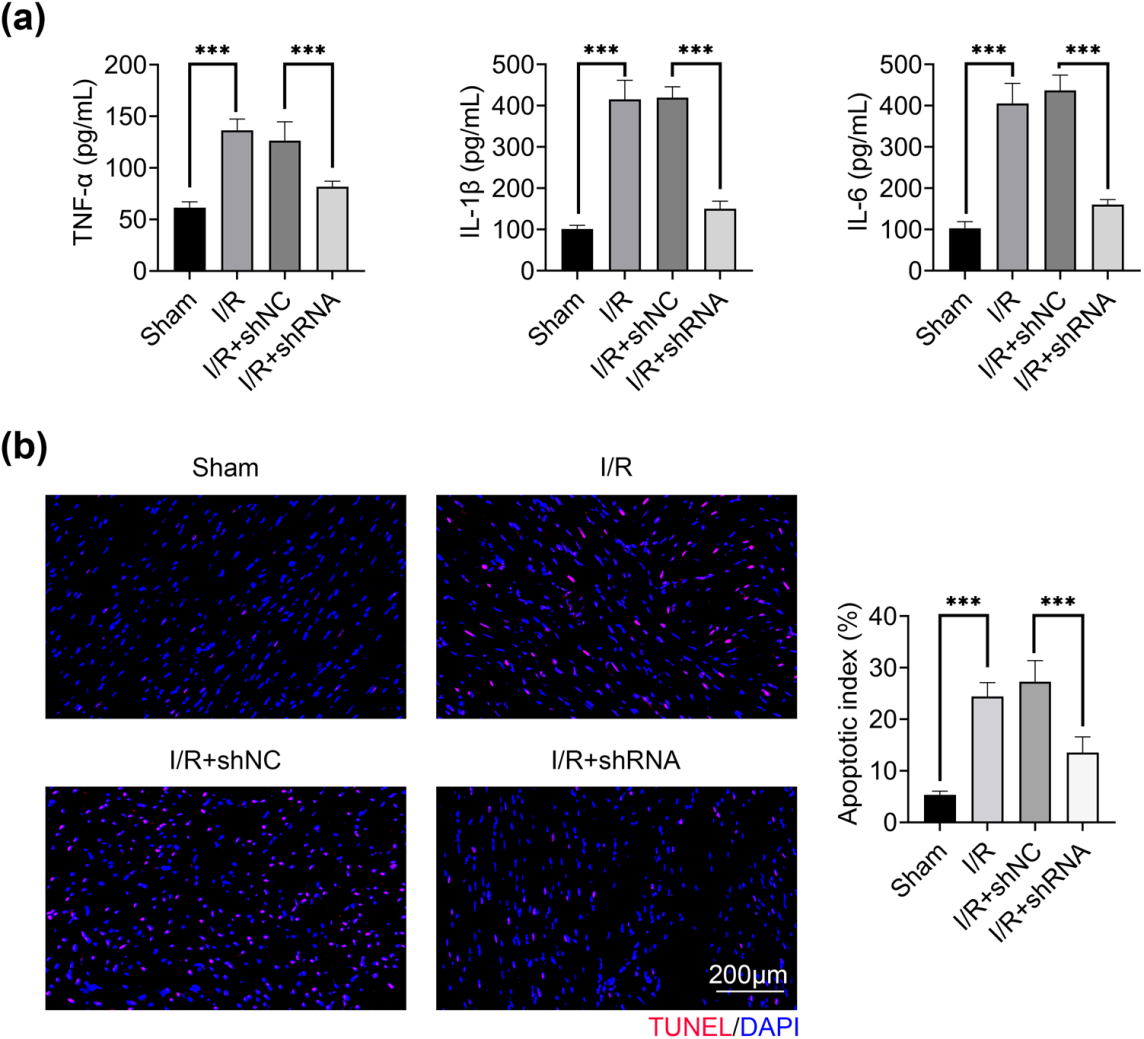
Knockdown of HAGLR prevented inflammation and cell apoptosis induced by I/R. (a) The levels of TNF-α, IL-1β and IL-6 in blood in I/R and I/R + shRNA groups were assessed by ELISA. ^***^ *p* < 0.001. Data were expressed as mean ± SD of three independent experiments. (b) TUNEL and DAPI staining were used to detect cell apoptosis. *Left*: representative photos of myocardial tissue stained by TUNEL and DAPI staining. *Right*: apoptotic index of myocardial tissue of I/R and I/R + shRNA groups. ^***^ *p* < 0.001. Data were expressed as mean ± SD of three independent experiments.

Next, effects of I/R and HAGLR on inflammation and cell apoptosis were evaluated by ELISA and TUNEL staining. I/R dramatically induced inflammatory response with a significant increase in TNF-α, IL-1β and IL-6 in the blood of I/R mice, which was completely inhibited by the knockdown of HAGLR (Figure 2a). I/R promoted cell apoptosis, and knockdown of HAGLR significantly suppressed cell apoptosis induced by I/R (Figure 2b). These observations demonstrated that knockdown of HAGLR could prevent inflammation and cell apoptosis induced by I/R.

### 3.3 HAGLR targeted and regulated the expression of miR-133a-3p

**Figure 3:**
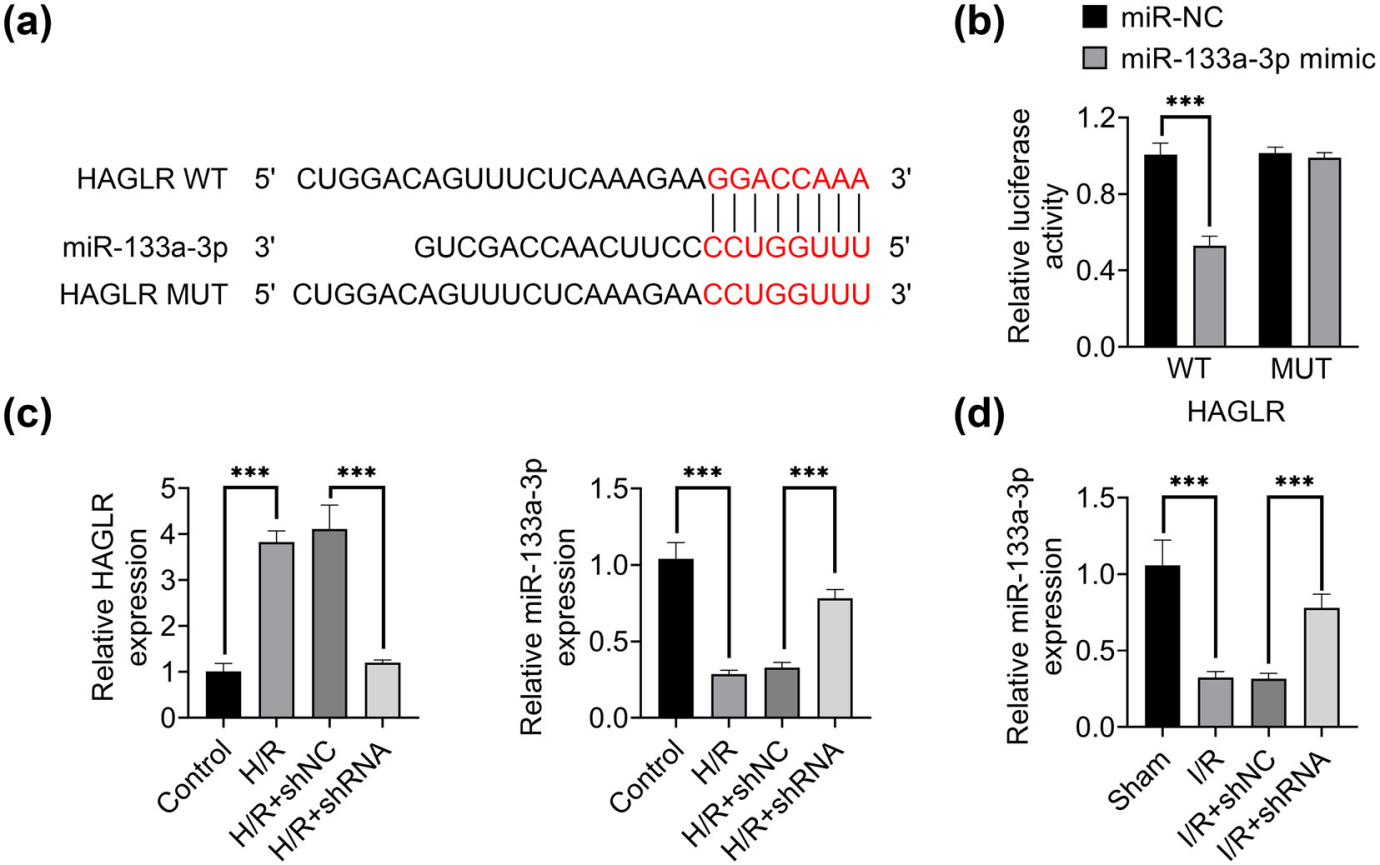
HAGLR targeted and regulated the expression of miR-133a-3p. (a) The binding site of HAGLR to miR-133a-3p was predicted based on the LncBase database. (b) Luciferase reporting system was used to detect the relative luciferase activity in HAGLR wild type (WT) and mutant (MUT) transfected with miR-NC or miR-133a-3p mimic. ^***^ *p* < 0.001. Data were expressed as mean ± SD of three independent experiments. (c) qRT-PCR was used to assess the expression of HAGLR and miR-133a-3p in H9C2 cells treated by H/R and HAGLR knockdown (H/R + shRNA). ^***^ *p* < 0.001. Data were expressed as mean ± SD of three independent experiments. (d) qRT-PCR was used to assess the expression of miR-133a-3p in myocardial tissue of I/R and I/R + shRNA mice. ^***^ *p* < 0.001. Data were ex*p*ressed as mean ± SD of three independent experiments.

Then, the molecular mechanism of HAGLR alleviating myocardial I/R injury was investigated. Based on the LncBase database, the binding site of HAGLR to miR-133a-3p was predicted (Figure 3a). Luciferase reporter assay revealed that HAGLR could interact with miR-133a-3p (Figure 3b). Hypoxia/reoxygenation (H/R) injury was used to mimic I/R in H9C2 rat cardiomyoblast cells. H/R induced significantly increased expression level of HAGLR but decreased the expression level of miR-133a-3p (Figure 3c). Knockdown of HAGLR not only prevented the increase of HAGLR expression induced by H/R but also prevented the decline of miR-133a-3p expression (Figure 3c). I/R caused a significantly decrease in miR-133a-3p expression in I/R mice, which could be rescued by the knockdown of HAGLR (Figure 3d). These data demonstrated that HAGLR could target and negatively regulate the expression of miR-133a-3p.

### 3.4 HAGLR modulated MAPK1 expression by targeting miR-133a-3p

**Figure 4:**
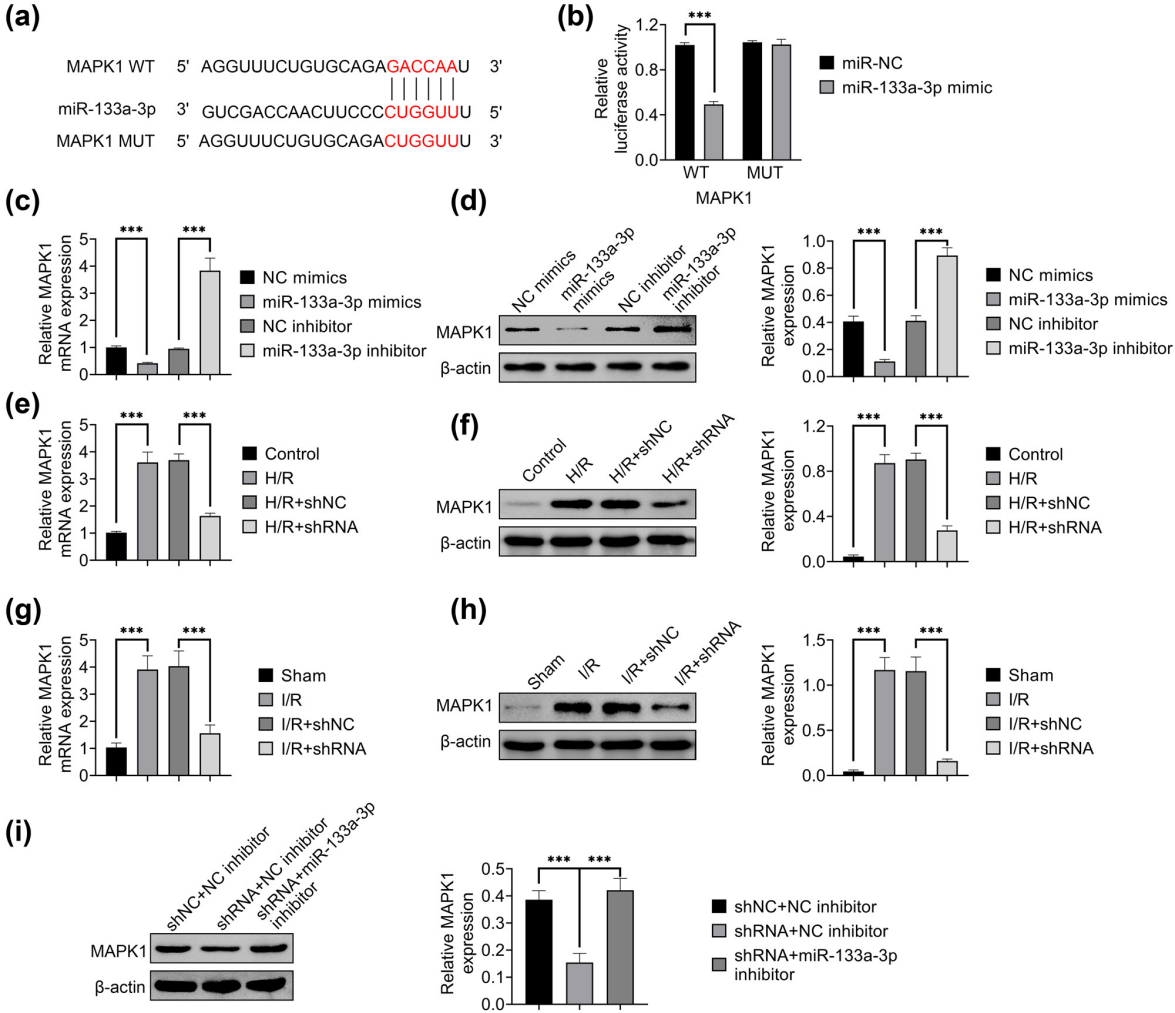
HAGLR modulated MAPK1 expression by targeting miR-133a-3p. (a) The binding site of miR-133a-3p to MAPK1 was predicted with ENCORI website. (b) Luciferase reporting system was used to detect the relative luciferase activity in MAPK1 wild type (WT) and mutant (MUT) transfected with miR-NC or miR-133a-3p mimic. ^***^ *p* < 0.001. Data were expressed as mean ± SD of three independent experiments. (c) qRT-PCR was used to assess the relative mRNA expression of MAPK1 in H9C2 cells transfected with miR-133a-3p mimic or treated by miR-133a-3p inhibitor. ^***^ *p* < 0.001. Data were expressed as mean ± SD of three independent experiments. (d) Western blotting was used to detect the protein expression of MAPK1 in H9C2 cells transfected with miR-133a-3p mimic or treated by miR-133a-3p inhibitor. ^***^ *p* < 0.001. Data were ex*p*ressed as mean ± SD of three independent experiments. (e) qRT-PCR was used to assess the relative mRNA expression of MAPK1 in H9C2 cells treated by H/R and H/R + shRNA. ^***^ *p* < 0.001. Data were expressed as mean ± SD of three independent experiments. (f) Western blotting was used to detect the protein expression of MAPK1 in H9C2 cells treated by H/R and H/R + shRNA. ^***^ *p* < 0.001. Data were expressed as mean ± SD of three independent experiments. (g) qRT-PCR was used to assess the expression of MAPK1 in myocardial tissue of I/R and I/R + shRNA mice. ^***^ *p* < 0.001. Data were expressed as mean ± SD of three inde*p*endent experiments. (h) Western blotting was used to detect the protein expression of MAPK1 in myocardial tissue of I/R and I/R + shRNA mice. ^***^ *p* < 0.001. Data were expressed as mean ± SD of three independent ex*p*eriments. (i) Western blotting was used to detect the protein expression of MAPK1 in H9C2 cells transfected with HAGLR shRNA with or without miR-133a-3p inhibitor. ^***^ *p* < 0.001. Data were expressed as mean ± SD of three independent experiments.

The functional role of HAGLR targeting miR-133a-3p and its molecular mechanism were then investigated. The binding site of miR-133a-3p to MAPK1 was predicted with ENCORI website (Figure 4a). The suppression of luciferase activity by the overexpression of miR-133a-3p was rescued by MAPK1 mutant, revealing that miR-133a-3p could interact with MAPK1 (Figure 4b). The relative expression of MAPK1 was significantly inhibited by the overexpression of miR-133a-3p but enhanced by miR-133a-3p inhibitor (Figure 4c and d). H/R treatment dramatically increased the relative expression of MAPK1, which was prevented by the knockdown of HAGLR in H9C2 rat cardiomyoblast cells (Figure 4e and f). The relative expression of MAPK1 was significantly increased in I/R mice, which was prevented by the knockdown of HAGLR (Figure 4g and h). The relative expression of MAPK1 was significantly inhibited by the knockdown of HAGLR, which could be rescued by miR-133a-3p inhibitor (Figure 4i). These results indicated that HAGLR could modulate MAPK1 expression by targeting miR-133a-3p.

### 3.5 HAGLR was involved in regulating inflammation and cell apoptosis induced by H/R

**Figure 5:**
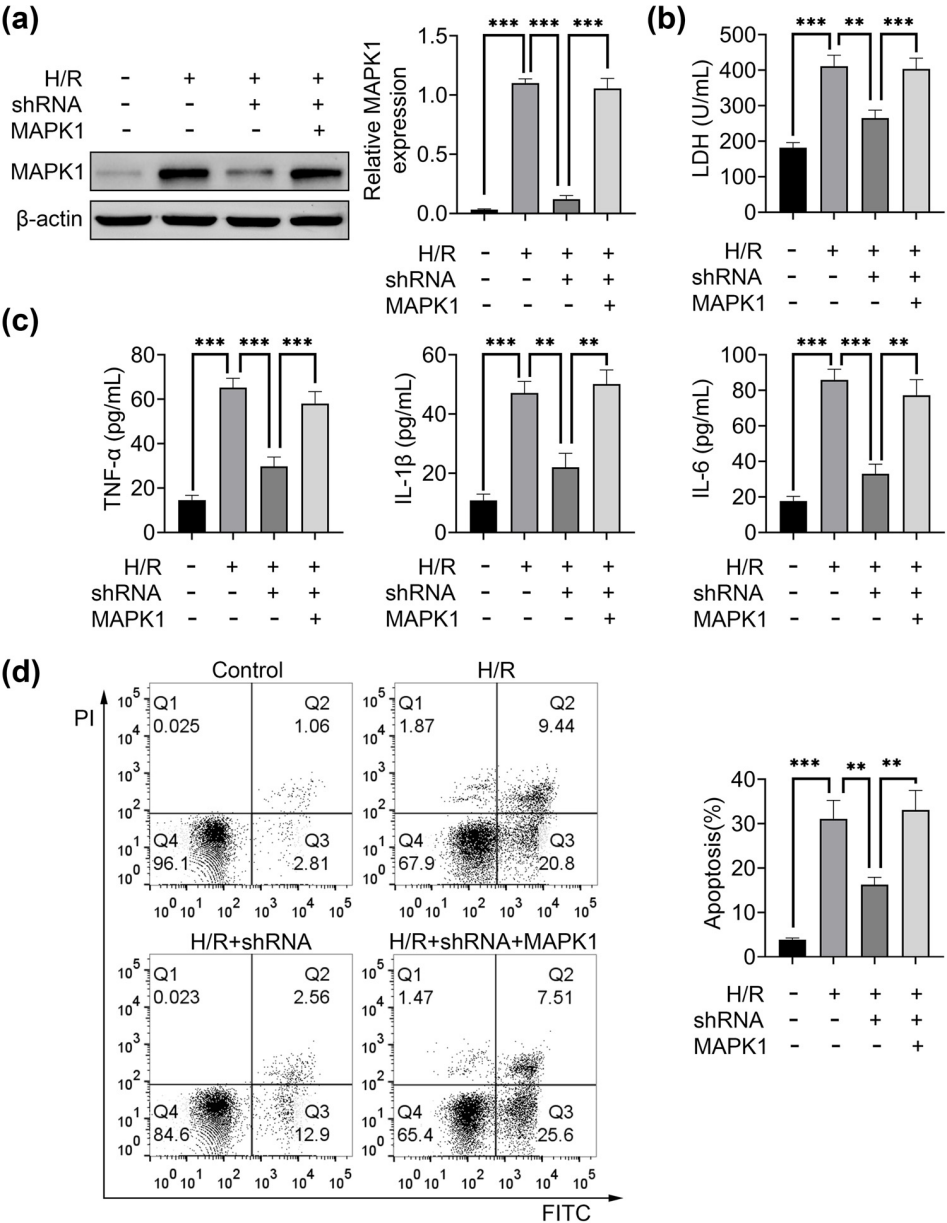
HAGLR was involved in regulating inflammation and cell apoptosis induced by H/R. (a) Western blotting was used to assess the relative protein expression of MAPK1 in H9C2 cells treated by H/R, knockdown of HAGLR (shRNA) and overexpression of MAPK1 (MAPK1). ^***^ *p* < 0.001. Data were expressed as mean ± SD of three independent experiments. (b) The LDH level in the culture medium with H9C2 cells treated by H/R, shRNA and MAPK1. ^**^ *p* < 0.01 and ^***^ *p* < 0.001. Data were ex*p*ressed as mean ± SD of three independent experiments. (c) ELISA was used to assess the levels of TNF-α, IL-1β and IL-6 of H9C2 cells treated by H/R, shRNA and MAPK1. ^**^ *p* < 0.01 and ^***^ *p* < 0.001. Data were expressed as mean ± SD of three independent experiments. (d) Flow cytometry was used to detect the cell apoptosis ratio of H9C2 cells treated by H/R, shRNA and MAPK1. ^**^ *p* < 0.01 and ^***^ *p* < 0.001. Data were expressed as mean ± SD of three independent ex*p*eriments.

H/R treatment enhanced MAPK1expression in H9C2 cells, which was comparable to the overexpression of MAPK1 (Figure 5a). Knockdown of HAGLR counteracted the effect of H/R treatment on MAPK1 expression (Figure 5a). H/R treatment and overexpression of MAPK1 promoted the release of LDH from H9C2 cells into culture medium, which was significantly suppressed by the knockdown of HAGLR (Figure 5b). H/R treatment and overexpression of MAPK1 dramatically increased the expression levels of TNF-α, IL-1β and IL-6, which was inhibited by the knockdown of HAGLR (Figure 5c). H/R treatment and overexpression of MAPK1 induced cell apoptosis, which was blunted by the knockdown of HAGLR (Figure 5d). These data suggested that HAGLR was involved in regulating inflammation and cell apoptosis induced by H/R.

## 4 Discussion

Acute myocardial infarction is a leading cause of morbidity worldwide [1,19]. Despite substantial progress in prognosis for myocardial infarction, the reperfusion of ischemic myocardium can induce myocardial reperfusion injury [2]. However, the underlying mechanisms responsible for myocardial I/R injury are still elusive and need further investigation. Unraveling the involved molecular mechanisms will provide therapeutic targets for myocardial I/R injury.

lncRNA is involved in the regulation of myocardial ischemia. For instance, lncRNA PEAMIR attenuated apoptosis and inflammatory response induced by I/R [13]. However, whether HAGLR plays a role in myocardial I/R injury and the underlying molecular mechanism have not been explored yet. miRNA participated in many pathophysiological developments and diseases [16,20]. lncRNA emerged as competitive endogenous RNAs and bind to miRNAs, resulting in the release of mRNAs, and, thus, lncRNA was able to modulate gene expression at post-transcriptional level [15]. miR-133a-3p possessed multiple functional roles in many physiological processes including myoblast proliferation [17]. A circular transcript of *ncx1* gene has been demonstrated to regulate cardiomyocyte apoptosis and ischemic myocardial injury by targeting miR-133a-3p [18]. It has shown that lncRNA MALAT1 modulated I/R injury by sponging miRNA-133a-3p [21]. Pentoxifylline alleviated I/R injury through the modulation of the expression of lncRNA-00654-miR-133a-SOX5 mRNA in rat hearts [22]. CircHelz activated NLRP3 inflammasome by sponging miR-133a-3p to promote myocardial injury in mouse hearts [23]. However, the molecular mechanism of miR-133a-3p modulating myocardial I/R injury remains to be further investigated, and whether lncRNA HAGLR could modulate I/R injury via miR-133a-3p was unknown.

In this work, it has shown that HAGLR expression was dramatically increased by I/R *in vivo* or H/R *in vitro*. Knockdown of HAGLR could alleviate myocardial I/R injury. I/R-induced inflammatory response and cell apoptosis were prevented by the knockdown of HAGLR. This study found that HAGLR could target miR-133a-3p and regulate the expression of miR-133a-3p. H/R treatment led to the declined expression of miR-133a-3p, which was rescued by the knockdown of HAGLR. MAPK1 expression was enhanced by I/R *in vivo* or H/R *in vitro*, which could be prevented by the knockdown of HAGLR. Overexpression of miR-133a-3p inhibited MAPK1 expression, which was attenuated by miR-133a-3p inhibitor. HAGLR could modulate MAPK1 expression by targeting miR-133a-3p. H/R treatment and overexpression of MAPK1 induced inflammatory response and cell apoptosis, which was blunted by knockdown of HAGLR. These data suggested that HAGLR regulated myocardial injury induced by I/R through miR-133a-3p/MAPK1 axis.

In summary, the present work demonstrated HAGLR promoting myocardial I/R injury by inhibiting miR-133a-3p, thus promoting MAPK1 expression. This work found that lncRNA HAGLR could regulate myocardial I/R injury and uncovered the underlying molecular mechanism. HAGLR might serve as a therapeutic target for treating the myocardial I/R injury.
